# My journey into mental healthcare as an international medical graduate (IMG)

**DOI:** 10.1192/bji.2021.25

**Published:** 2022-05

**Authors:** Süreyya Melike Toparlak

**Affiliations:** Psychiatry and Psychotherapy Resident, Clinic for Addiction Medicine and Addictive Behaviour, Center for Mental Health, Bad Cannstatt Hospital, Stuttgart Clinic, Germany; Oxford Health NHS Foundation Trust, Warneford Hospital, UK. Email sureyya.toparlak@oxfordhealth.nhs.uk

**Keywords:** Education and training, immigration, international medical graduate (IMG), psychiatry and psychotherapy, Black, Asian and minority ethnic (BAME)

## Abstract

Over half of doctors who joined the General Medical Council's (GMC) medical register in the UK in 2020 were from Black, Asian and minority ethnic (BAME) background, and I am one of them. Having experienced clinical settings across three different countries, I think my journey deserves to be shared with others as a unique experience. My story will help medical students and early career doctors have a general idea of different clinical settings and hopefully will encourage them to chase their dreams. I hope to inspire people.

The 2020 *State of Medical Education and Practice in the UK* report by the GMC^1^ showed an increase in the diversity and importance of international medical graduates (IMGs) in the National Health Service (NHS): 54% of the doctors joining the register in 2020 were from Black, Asian and minority ethnic (BAME) backgrounds. I am one of them. I graduated from Istanbul University Faculty of Medicine. I possess the unique honour of having full registration with a licence to practise as a doctor in three different countries, namely Turkey, Germany and the UK. Many people have contacted me asking about my experience working abroad as a doctor. By putting my journey and my objective and subjective observations of the work atmosphere into words, I am sure that others will benefit from my experience.

## Turkey

According to a 2018–2020 study by the Turkish Medical Association,^2^ the number of doctors who requested a Good Standing Certificate has increased steadily. This certificate is necessary to apply for registration to work as a doctor outside the country. In November 2019, this number was 906, whereas it was just 59 in 2012. There are many reasons why Turkish medical students and doctors move to another country to practise medicine. These include the increased violence by patients and their relatives against healthcare professionals, the performance-based system, the intensity of emergency services, an insecure and unsafe workplace and anti-democratic practices.

After graduating from medical school, every doctor has to carry out compulsory service in accordance with the law. The government decides in which areas doctors will be working, according to need. Mollahaliloglu et al^3^ highlighted physician scarcity and overwork as issues in this obligatory role. Owing to this obligation, I worked in the emergency department in a state hospital in Turkey. There, doctors have a minimum of eight 24 h shifts per month. The number of patients varied from 800 to 1000 per day. It was quite challenging to work under those circumstances.

## Choosing psychiatry

I have always been interested in interpersonal relationships, people's lives, and the relationship between the mind, body and emotions. After attending just one psychiatry lecture at university, I chose psychiatry as my future career path. The lecture was about the intriguing case presentation of a patient with self-harm, depression and family issues. I was mesmerised by how our professor took the patient’s history and then analysed him without any imaging techniques. I agree with Professor Nandini Chakraborty when she said that ‘It takes very special people to understand the person behind a patient, to listen to what is unsaid, see what is unapparent’.^4^ As she continued, ‘I find psychiatrists among the most self-reflective, compassionate, always balancing the ethical [sic.] dilemmas professionals. […] We were drawn to this profession for a reason’.^5^ Her sentiment explains very well why I chose psychiatry, why I was drawn to the profession and why I am extremely passionate about my job.

## Germany

To register as a doctor in Germany, I had to pass three exams. First, I took a German language test. Second, an occupational German test (*Fachsprachprüfung*). This exam consists of three parts: a doctor–patient conversation (anamnesis), documentation and a doctor–doctor conversation. After that, one is granted a temporary working permit (*Berufserlaubnis*). The last exam is the German medical exam. It consists of clinical and oral practical parts under the lead of three medical doctors from different specialties. It grants one the permanent right to work in the country as a doctor. Following this, and having worked a certain number of years in the country depending on personal circumstances, one is allowed to work in other EU countries (excluding the UK).

Regarding my experience and observations as a psychiatry and psychotherapy trainee in Germany, I worked in two different cities. One was in Lower Saxony in the north-west and the other was in Baden-Württemberg in the south-west of the country. For me, one of the main advantages of working in Germany was that they offer psychotherapy training to all trainees in psychiatry. I could observe and experience different types of therapy methods in Germany. These include art therapy, body therapy, drama therapy, gardening groups, cooking groups, ward sports groups, ergotherapy (occupational therapy), and also morning and evening ward rounds, which include discussion about current issues and problems, the daily routine and group and leisure activities. I joined in one of the ergotherapy sessions and I learned how to make a bracelet with the patients, which I still keep as a remembrance. Apart from believing in the healing power of art, I have a keen interest in poetry, Latin dance and art therapy, and I play many instruments. Therefore, I call myself a ‘psychiARTist’. I aim to use my talents to benefit my patients and to provide better mental healthcare. It was amazing to witness how team members analyse patients’ paintings/drawings under the lead of art therapists in multidisciplinary team (MDT) meetings. Another great thing was that hospitals support compulsory external training, such as self-psychotherapy. The entire psychotherapy and psychiatry training takes 5 years in total: 4 years in mental health services and 1 year in a neurology department. Some of the compulsory tasks are family meetings, autogenic or hypnotherapy training, psychotherapy sessions as well as Balint groups.

## The UK

To work in the UK, I had to follow similar steps as in Germany. So, I took a set of exams. These were the Occupational English Test (OET) and the two Professional and Linguistic Assessments Board (PLAB) tests, PLAB 1 and PLAB 2. While taking these exams, I was lucky enough to complete a clinical attachment in the Basildon Mental Health Unit and a European Federation of Psychiatric Trainees (EFPT) Exchange Programme at Bethlem Royal Hospital in South London and Maudsley NHS Foundation Trust during a holiday. I shared this amazing experience in the bumper issue of *The Registrar (*https://www.rcpsych.ac.uk/docs/default-source/training/training/ptc/the-registrar-bumper-issue-final.pdf?sfvrsn=fda558b7_2*)*. I was extremely fortunate to make a clinical attachment in Essex Partnership University NHS Foundation Trust (EPUT) under the supervision of the co-founder of Hip Hop Psych, Dr Akeem Sule. I learned a lot from him in such a short time, such as psychiatric assessment, the role of MDT members and importance of effective communication. After my experience in EPUT, I was sure that I wanted to move to the UK to work as a psychiatrist. Thanks to Professor Rashid Zaman’s invitation, I made an oral presentation at the Cambridge International Conference on Mental Health 2019 (https://www.cmhr-cu.org/Conference2019), where I met many international psychiatrists and exchanged ideas with them. My supervisors and colleagues helped me to extend my knowledge about the training system in the UK. I liked that there is always an opportunity for us to improve the care we provide, with audits and quality improvement projects. Moreover, I was impressed by the diversity among healthcare professionals. Every psychiatrist I had met in the UK has been open-minded, honest, cheerful, encouraging, kind, empathetic, caring and helpful. I felt very welcomed during my time in the UK. I was invited to official teaching events, where we always had delicious food and excellent speakers. Furthermore, I built long-lasting friendships in such a short time.

The training pathway in the UK after graduation seems to be longer than in Turkey and Germany, but there are many helpful online resources to consult when one is lost.

To conclude, these experiences enriched me, both professionally and personally. They broadened my horizon. I look forward to my next post in the Oxford Health NHS Foundation Trust, where I feel very welcomed even before starting. I have no doubt that I will learn a lot but at the same time teach others. This unique experience helped me to learn NHS values by heart by living them. I am sure that I will make a valuable contribution to the NHS, bringing with me my enthusiasm and international experiences.

## Data Availability

Data availability is not applicable to this article as no new data were created or analysed in this study.
